# Mitigating Fox Predation on Freshwater Turtle Nests: Comparing Effectiveness of Three In Situ Protection Methods

**DOI:** 10.1002/ece3.72121

**Published:** 2025-09-08

**Authors:** Christina Hunter, Deborah S. Bower, Richard A. Peters, Ricky‐John Spencer, Ligia Pizzatto, James U. Van Dyke

**Affiliations:** ^1^ Department of Ecological, Plant & Animal Sciences Centre for Freshwater Ecosystems, School of Agriculture, Biomedicine and Environment, La Trobe University, Albury‐Wodonga Campus West Wodonga Victoria Australia; ^2^ School of Environmental and Rural Science, University of New England Armidale New South Wales Australia; ^3^ Department of Ecological, Plant & Animal Sciences School of Agriculture, Biomedicine and Environment, La Trobe University, Bundoora Campus Bundoora Victoria Australia; ^4^ Western Sydney University, School of Science Penrith New South Wales Australia

**Keywords:** anti‐predator fencing, endangered species, floating island, invasive species, predator management, recruitment

## Abstract

Freshwater turtles in the Murray‐Darling Basin (MDB), Australia, have declined since the 1970s. Intense nest predation by introduced foxes likely contributes to these declines, disrupting juvenile recruitment needed to sustain populations. Traditional lethal control methods, such as baiting and shooting, have proven inadequate, highlighting the need for innovative conservation strategies. We tested three nest protection methods—fenced nesting beaches, artificial floating islands (artificial nesting habitat), and individual mesh covers—for reducing fox predation. Using artificial turtle nests across protected and unprotected plots, we monitored nest predation with remote cameras and confirmed nest status through excavation. On average, nest predation was lowest on artificial islands (17%), followed by fences (37%) and mesh (40%). All protection methods significantly reduced depredation compared to unprotected controls (85% destroyed). Unprotected nests were almost exclusively depredated by foxes, while protected nests saw more predation from native animals. Native predator species did not differ among protection treatments. Our findings underscore the potential for artificial floating islands as a valuable conservation tool. Further research into optimizing nest protection and understanding ecological impacts is critical for improving recruitment and reversing declines of freshwater turtle species.

## Graphical abstract

Floating islands provide the best protection for freshwater turtle nests, compared to fenced nesting beaches and individually protected nests. However, all three methods are better than leaving nests unprotected.

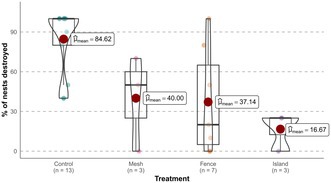

## 1 Introduction

Nest predation is a significant threat to the reproductive success of many oviparous species and is a common cause of egg and juvenile mortality (Congdon et al. 1983; Menezes and Marini 2017; Ricklefs 1969; Schwanz et al. 2010). Introduced species can increase nest predation beyond historic rates by thriving in new environments and exploiting native species that have not evolved defenses against them (Banks and Dickman 2007; Doherty et al. 2016; Fea et al. 2021). High mortality rates in young age classes are a significant problem for threatened species, as small population sizes, restricted distributions, and additional environmental pressures heighten their sensitivity to lost juvenile recruitment (Doherty et al. 2015; Webb et al. 2002). Developing effective, evidence‐based methods to reduce nest predation is crucial to prevent current and predicted population declines driven by recruitment failure.

Lethal control methods like shooting and poison baiting are commonly used for controlling introduced predators in Australia (Doherty et al. 2015; Doherty and Ritchie 2017; Saunders et al. 2010). While these methods may provide some short‐term benefits (Christiansen and Gallaway 1984; Fulton and Ford 2001; Smith et al. 2010), they only reduce the number of predators (i.e., numerical response). Lethal controls do not account for the predator functional responses, which describe how the intake of an individual predator might change based on prey availability (Holling 1959; Solomon 1949). For highly efficient mammalian predators, such as raccoons ( *Procyon lotor* ) and foxes ( *Vulpes vulpes* ), predation rates by a small number of individuals can remain high despite large reductions in aggregate predator densities (Ratnaswamy et al. 1997; Spencer et al. 2017). To address the functional response, alternative nest protection methods are more effective by reducing predator access to nests using exclusion devices or barriers. For example, protective cages and guards on nest boxes have improved nesting success in numerous ground‐nesting and arboreal bird species (Gautschi et al. 2024; Stojanovic et al. 2019), and exclusion fencing has been used to create predator‐free havens for many threatened Australian mammal species (Legge et al. 2018).

Turtles are a highly threatened vertebrate group often exposed to high nest predation rates over their extended incubation periods (Lovich et al. 2018), offering a model system to study the effectiveness of various nest protection methods. Turtles are likely afforded some resilience to high egg and juvenile mortalities through their long lifespans, iteroparity, and relatively high fecundity (Congdon et al. 1993; Mullin et al. 2023; Spencer and Thompson 2005). However, extended periods of severely limited recruitment can jeopardize population stability and place turtle populations at greater risk of extinction as there is no replacement of older adults once they die (Lovich et al. 2018; Spencer 2018; Spinks et al. 2003). In Australia, foxes are a major threat to turtles, with nest predation rates routinely exceeding 90% (Congdon et al. 1987; Terry et al. 2023; Thompson 1983b). In Australia's Murray‐Darling Basin, some populations of *Emydura macquarii* and *Chelodina longicollis* have experienced reductions of up to 69%–91% since the 1970s; declines which were likely initiated by drought and exacerbated by heavy nest predation from invasive foxes (Chessman 2011; Thompson 1983b). Lethal methods are often ineffective for controlling foxes (Saunders et al. 2010), especially when targeting turtle protection (Robley et al. 2018). Few studies have directly compared the effectiveness of different predator mitigation strategies for turtle nests, limiting evidence‐based recommendations on the most appropriate method for long‐term recruitment improvement. Thus, the development and testing of new methods are needed to effectively protect turtle nests and curb declines.

Our study compares the effectiveness of three non‐lethal nest protection methods: wire mesh placed over individual nests, fenced nesting beaches, and artificial floating islands (introduced nesting habitat). Securing mesh grids over nests is a popular and inexpensive protection method that has successfully reduced nest predation in several turtle species (Bougie et al. 2020; Campbell et al. 2020; Lei and Booth 2017; O'Connor et al. 2017; Yerli et al. 1997). Exclusion fencing is less widely used but has effectively excluded foxes from western swamp turtle (*Pseudoemydura umbrina*) and western saw‐shelled turtle ( *Myuchelys bellii* ) nests in Australia (Guyot and Kuchling 1998; Streeting et al. 2023), and raccoons from map turtle (*Graptemys* spp.) nests in the USA (Geller 2012). Artificial floating islands have provided safe nesting habitat for numerous waterbird species (Hancock 2000; Manikowska‐Ślepowrońska et al. 2022; McIntyre and Mathisen 1977; Nakamura and Mueller 2008), and, when vegetated, may additionally provide emerging hatchling turtles with food resources and refuge habitat in the root structures below, as seen in fish (Karstens et al. 2021; Nakamura and Mueller 2008). Both the mesh grids and fenced nesting beaches are designed to physically block foxes, while artificial floating islands offer nest protection based on the assumption that foxes will not swim to reach them—an assumption that requires testing. While these protection measures may effectively exclude foxes, research is lacking on how native predators affect turtle nests in the absence of invasive species. Turtle eggs are high in lipids and proteins (Booth 2003), making them a sought‐after resource for many native predators including monitor lizards (*Varanus* spp.; Georges and Kennett 1989), ravens (*Corvus* spp.; Baggiano 2012), rakali ( *Hydromys chrysogaster* ) (Thompson 1983b) and echidnas (Robinson et al. 2024). By removing competition from foxes, nest protection methods may inadvertently attract or concentrate native predators with similar capacities for nest predation (Chessman 2021; Stantial et al. 2021) and ultimately fail to provide effective protection and improve recruitment outcomes.

Data on novel nest protection methods are still required to assess their cost‐effectiveness and to better understand the extent of native predation in the absence of foxes. Here, we aimed to address the specific questions of (1) which nest protection method is most effective at preventing fox predation, and (2) does the composition of predator species differ between different protection treatments? To address these questions, we created artificial nests and compared the rates of predation between unprotected nests and those protected by mesh, exclusion fencing, and artificial floating islands. Remote camera footage was used to identify and quantify the predatory species responsible for each nest depredation event on both unprotected and protected nests.

## 2 Study Area

**FIGURE 1:**
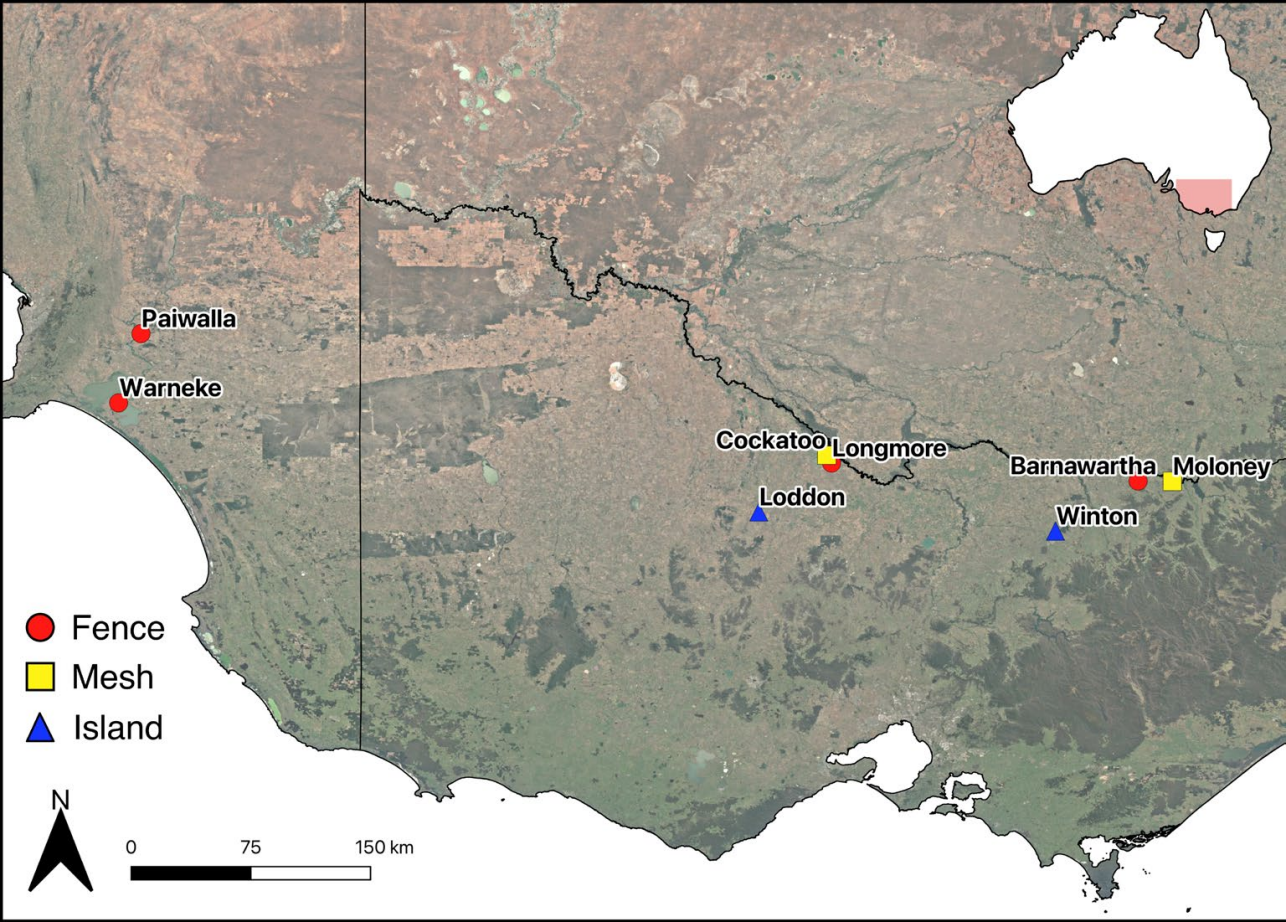
Map of all study sites where nest plots protected by mesh, exclusion fencing, and artificial floating islands were established across Victoria and South Australia, Australia, in 2023–2024. Sites include Barnawartha (−36.09969, 146.62305), Cockatoo (−35.91285, 144.35736), Longmore (−35.96928, 144.39391), Loddon (−36.32531, 143.86485), Moloney (−36.10249, 146.87221), Paiwalla (−35.03198, 139.37398), Warneke (−35.53079, 139.20901), and Winton (−36.46479, 146.02267).

The field experiment was conducted at eight sites across north‐central Victoria and south‐eastern South Australia (Figure 1). These sites were a combination of lagoons, anabranches, constructed reservoirs, and natural wetlands connected to the broader Murray‐Darling Basin. Sites were selected for their continuous habitat, characterized by open agricultural land, native grasslands, and riparian vegetation, as well as their proximity to active turtle nesting areas identified through TurtleSAT (www.turtle sat.org.au), a citizen science project that collects turtle sightings and nest data across Australia via a mobile app.

## 3 Methods

### 3.1 Field Experiment

Field research was conducted after the peak laying period (October to early December) of the two focal species, *E. macquarii* and *C. longicollis* , to prevent attracting additional foxes to incubating turtle nests and disrupting nesting females. Artificial nest plots were carefully situated to avoid turtle nests mapped in TurtleSAT whilst remaining within ~100 m of known nesting locations. Artificial nest plots were placed within similar soils as real nests to mimic turtle preferences at a given site, which included a range of hard, soft, and sandy soils all within ~200 m of the water's edge (Kennett et al. 2009; Petrov et al. 2018; Spencer and Thompson 2003). Nest plots were created between mid‐December 2023 and early January 2024 and buried over 3 months to resemble an average between the typical incubation periods of *E. macquarii* and *C. longicollis* (Kennett et al. 2009; Spencer 2002). At the end of the study period, the number of destroyed nests in a plot was determined through a combination of visual surveys, excavation of nests with a hand trowel, and inspection of remote camera images. All procedures were conducted in accordance with the approval from the La Trobe University Animal Ethics Committee (#AEC24017).

### 3.2 Plot Creation

**FIGURE 2:**
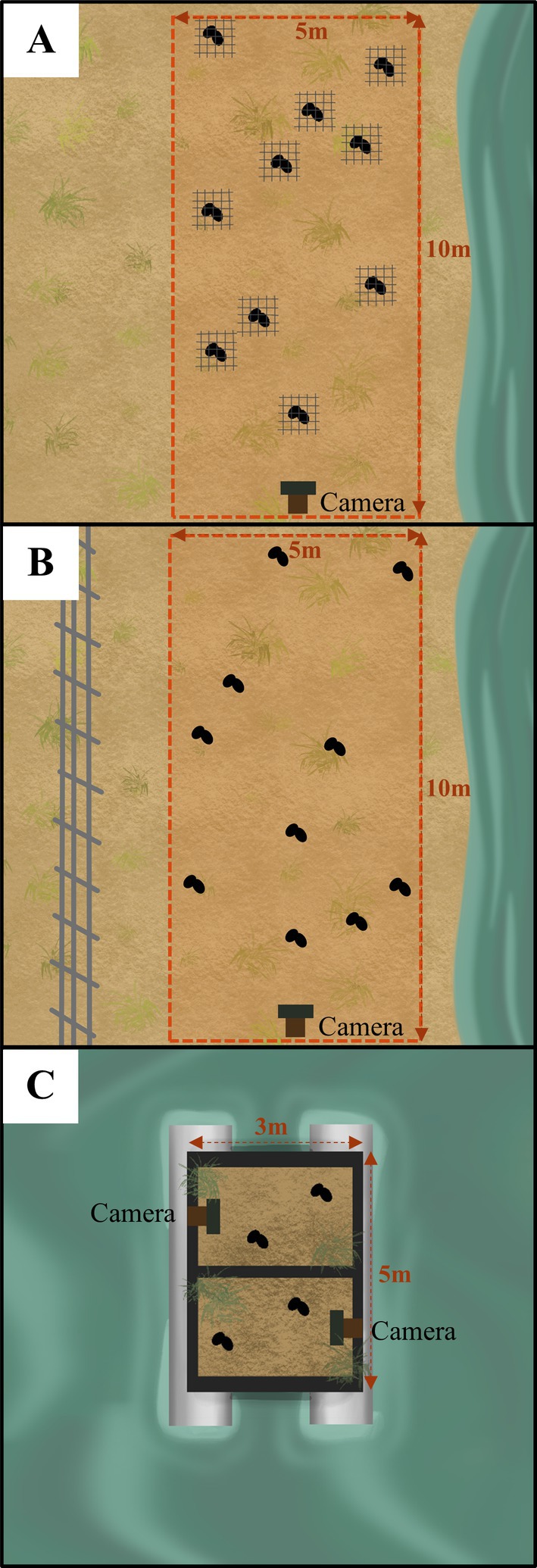
Setup of nest plots containing 10 nests under the mesh (A) and fenced (B) protection treatments, and nest plots containing four nests under the artificial island (C) treatment. On artificial islands, the four nests were distributed across two nesting tubs due to the island's design. However, each artificial island was considered a single nest plot, meaning one island corresponded to one nest plot.

Due to site‐specific limitations, including wetland size, area of existing fenced nesting beaches, and the availability and size of artificial floating islands, the number and arrangement of nest plots varied slightly across sites and treatments (Figure 2). However, all nest plots were constructed using the same standardized protocol, and these differences were accounted for in the statistical analysis. The design and construction of nest plots followed established methods used in previous studies assessing nest predation in freshwater turtles (e.g., Terry et al. 2023; Dawson et al. 2014).

### 3.3 Mesh

Mesh protection was used on the banks of two wetlands in northern Victoria, Australia. (Figure 1), and involved covering each of the 10 nests in a plot with a 50 cm × 50 cm square of wire chicken netting (1.2 mm diameter, 50 mm aperture) held in place by eight tent pegs at each corner and side. At the larger wetland (Moloney, Wodonga, VIC), two meshed plots and two corresponding control plots were created in late December 2023. Control plots were created within 100 m of the treatment plots and constructed immediately after the treatment plots. At the second, smaller wetland (Cockatoo, Gunbower VIC) only one treatment and one control plot were created in mid‐December 2023. Artificial nest plots measured 10 m × 5 m within the mesh treatments, with wooden stakes set at each corner and midpoint of each side of the plot. To capture the types of predators and date/time of nest raiding, one stake was fitted with an infrared, motion‐sensing remote camera (Campark T85) positioned downward and orientated to minimize glare, at a distance of at least 0.5 m from the closest nest. The camera was also aimed to ensure that its field of view captured all 10 nests in the plot. An electric drill and auger bit were used to dig 10 nest holes, each 150–200 mm deep, spaced at least 0.5 m apart and placed haphazardly within the plot (Figure 2). Large store‐bought chicken eggs were used as proxies for turtle eggs to avoid sacrificing real turtle eggs, consistent with previous studies (Dawson et al. 2014; Grosse et al. 2015; Terry et al. 2023). Two ~60 g chicken eggs were placed in each nest hole to represent an intermediate between the average mass of an *E. macquarii* and *C. longicollis* clutch (Kennett et al. 2009; Spencer and Thompson 2005; Thompson 1983a). Once buried, marker flags were positioned on nests and photos were taken at each corner and side of the plot to allow for nests to be later located for excavation by estimating their distance from the known points of the perimeter stakes and adjacent nests. All marker flags and non‐camera stakes were immediately removed after the construction of nest plots was complete.

### 3.4 Fence

Nests that were protected by fencing had 1 to 2 plots created inside fenced nesting beaches at four separate sites (Figure 1). Both control and treatment plots were created in late December 2023 at Longmore and Barnawartha, and in early January 2024 in Paiwalla and Warneke (Figure 1), with the creation of control plots occurring immediately after the construction of treatment plots at all sites. The fenced areas were purpose‐built to protect known turtle nesting areas between 2016 and 2023. Each fence was constructed primarily of Cyclone or generic anti‐predator fencing at least 2 m tall, with 0.5 m of skirting on the ground. The aperture of the fence mesh was 4 cm in diameter at most. The fence enclosed areas of varying sizes on three sides, where the fourth side was always open to an adjacent wetland. The wetland ends of the fencing extended into the wetland at least 3 m at all locations to prevent wildlife from easily walking around the ends of the fence and into the enclosed area. This arrangement allows turtles to access the fenced area from the wetland but prevents any direct land access. Although real turtle nests were not used, turtle access was considered in our design so the results from the artificial nests could be meaningfully applied to actual nesting scenarios. The smallest fenced site (Barnawartha, VIC) was only large enough to construct one plot containing 10 nests inside the fence, while the remaining larger fenced sites (Longmore, Gunbower VIC, Paiwalla SA, Warneke SA) all had two plots constructed, also containing 10 nests. An equal number of control plots with 10 nests in each were also created outside the fence, within 100 m of the fenced nests. The construction of nest holes, camera type, and camera setup in the fenced treatment were identical to those in the mesh treatment.

### 3.5 Artificial Floating Islands

Artificial floating islands (hereby AFIs) were deployed at two sites (Figure 1). One site had only one AFI (Winton, VIC), while the larger remaining site (Loddon, VIC) had two AFIs anchored ~50 m apart. Plot construction was modified under the island treatment to accommodate the size limitations of the AFIs (Figure 2). The floating islands were comprised of a 5 m × 3 m steel mesh platform supported by eight 150 mm diameter PCV tubes, sealed at both ends to ensure buoyancy. Two nesting boxes, each measuring 650 mm × 400 mm, were placed on top of the steel platform and filled with soil. Steel mesh frames wrapped in hessian jute (burlap) were attached to the sides of nesting tubs to create turtle access ramps, and coconut fiber was wrapped across all remaining exposed mesh. AFIs were deployed by kayaks and anchored using sand anchors and a 5 mm marine‐grade stainless steel cable.

Using the same protocol as above, four artificial nests were created on each of the three AFIs (two nests per nesting box) in mid‐December 2023, with remote cameras placed on opposite ends of the nesting tubs to capture predation events. To match the smaller size of the island plots, 5 m × 2.5 m control plots with five nests per plot were created on the nearest shore immediately following the construction of nests on artificial islands.

### 3.6 Statistical Analysis

#### 3.6.1 Nest Protection

We conducted all statistical analyses in the R statistical environment (v4.3.3; R Core Team 2021). To analyze the relationship between the nest protection treatment and the proportion of destroyed nests while accounting for variation between locations, we employed a Generalized Linear Mixed Model (GLMM) to model logistic regressions. The GLMM was fitted using the ‘glmmTMB’ function from the ‘glmmTMB’ package (Brooks et al. 2017). The GLMM compared the number of destroyed nests per plot out of the total number of nests within the plot as the response variable. The treatments (mesh, island, fence, control) were incorporated as categorical fixed effects, and the location of plots (out of eight potential sites) as a categorical random effect to account for potential correlation and variability within and across locations. The model was fitted with a binomial family and logit link function. Estimated marginal means were obtained using the ‘emmeans’ function in the ‘emmeans’ package (Lenth 2024). To assess the significance of the fixed effects (treatment), we performed Type III tests using the ‘anova’ function. Type III tests determine the significance of each treatment effect on nest predation rates, while accounting for the other factors in the model. Pairwise comparisons were then performed with Tukey adjustment to account for multiple testing.

#### 3.6.2 Predator Types

Data were obtained from camera images, which recorded the predator responsible for every predation event within each plot. Images from the remote cameras were manually reviewed, and predation events were recorded when an animal was observed either (a) holding an egg or egg fragments in or near its mouth above the nest cavity, or (b) inserting its snout or head into a nest cavity. Since the flags marking nest locations were removed after setup, images of predation events were manually overlaid against reference images of the flagged nests to confirm that animals were excavating artificial nests rather than disturbing random areas of ground. These observations were further validated by comparing them with the known depredation status of nests determined by excavating the nests at the end of the study period. From these images, we compiled data on predator occurrences for each nest plot, converted occurrence data into a dissimilarity matrix, and used Non‐metric Multidimensional Scaling (NMDS) with the ‘vegan’ and ‘dplyr’ packages in R (Oksanen et al. 2023; Wickham et al. 2023) to compare the types of predators raiding nests across protected and control plots. NMDS analysis visualizes differences in predator composition across treatment and control groups, with axes representing gradients of dissimilarity in predator composition and relative distances between points reflecting similarities or differences in predator composition. The NMDS was created using the ‘metaMDS’ function with a Euclidean distance. Differences in predator compositions between protection treatments and control groups were statistically tested using a PERMANOVA on the Euclidean distance matrix, which was based on the proportions of nest predation attributed to each predator. We then performed pairwise comparisons using the ‘pairwiseAdonis’ function to identify specific differences between treatment and control groups.

## 4 Results

### 4.1 Nest Protection

**FIGURE 3:**
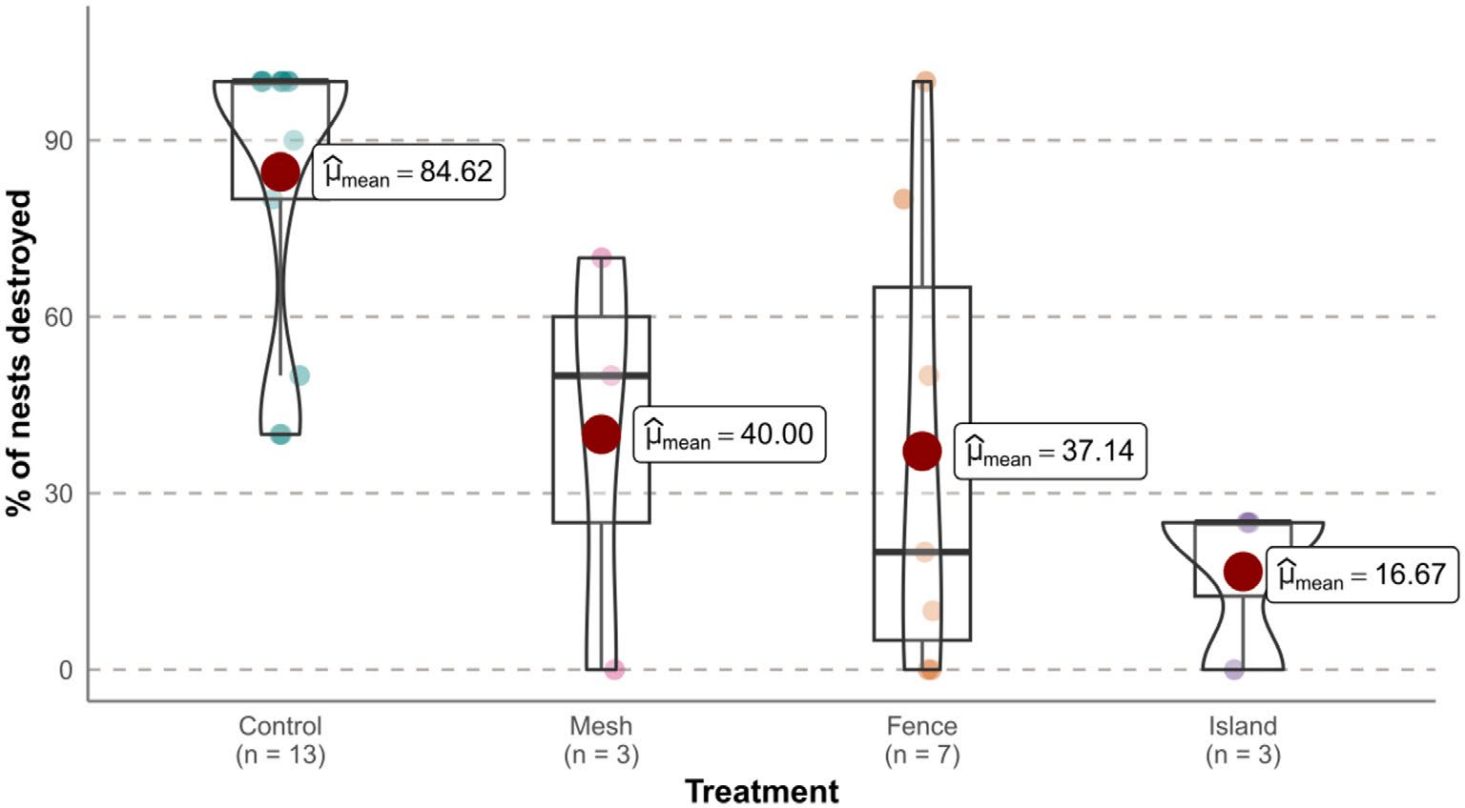
Violin plots with inset boxplots of the percentage of artificial freshwater turtle nests destroyed under each treatment in Victoria and South Australia, Australia 2023–2024. Individual sites are represented by each data point, with *n* = total number of sites within each treatment group. Mean values are indicated by red circles within each box plot.

On average, the control plots had the highest percentage of destroyed nests, with nest predation occurring at more than twice the rate of mesh and fence‐protected nests and five times the rate of nests on islands (Figure 3). The GLMM analysis showed that nest predation rates differed across the treatments (*F*_3,15_ = 19.16, *p* < 0.05). The mesh, fence, and island treatments all experienced less nest predation compared to the control, but there were no significant differences in depredation rates between the protection treatments (Figure 3, Table 1).

**TABLE 1:** Pairwise comparisons of freshwater turtle nest protection methods in Victoria and South Australia, Australia 2023–2024. Results based on the estimated marginal means extracted from the generalized linear mixed model (GLMM), including the magnitude and direction of difference between pairwise groups (estimate) and standard error (SE). Significance (*p* < 0.05) is indicated by asterisks.

Treatment	Estimate	SE	*t*	*p*
Control vs. Fence	3.20	0.53	6.04	< 0.001*
Control vs. Island	2.76	1.02	2.71	0.035*
Control vs. Mesh	3.99	1.10	3.64	0.002*
Fence vs. Mesh	0.79	1.10	0.72	0.890
Island vs. Mesh	1.23	1.53	0.80	0.853
Fence vs. Island	−0.44	1.15	−0.38	0.981

### 4.2 Predator Types

**FIGURE 4:**
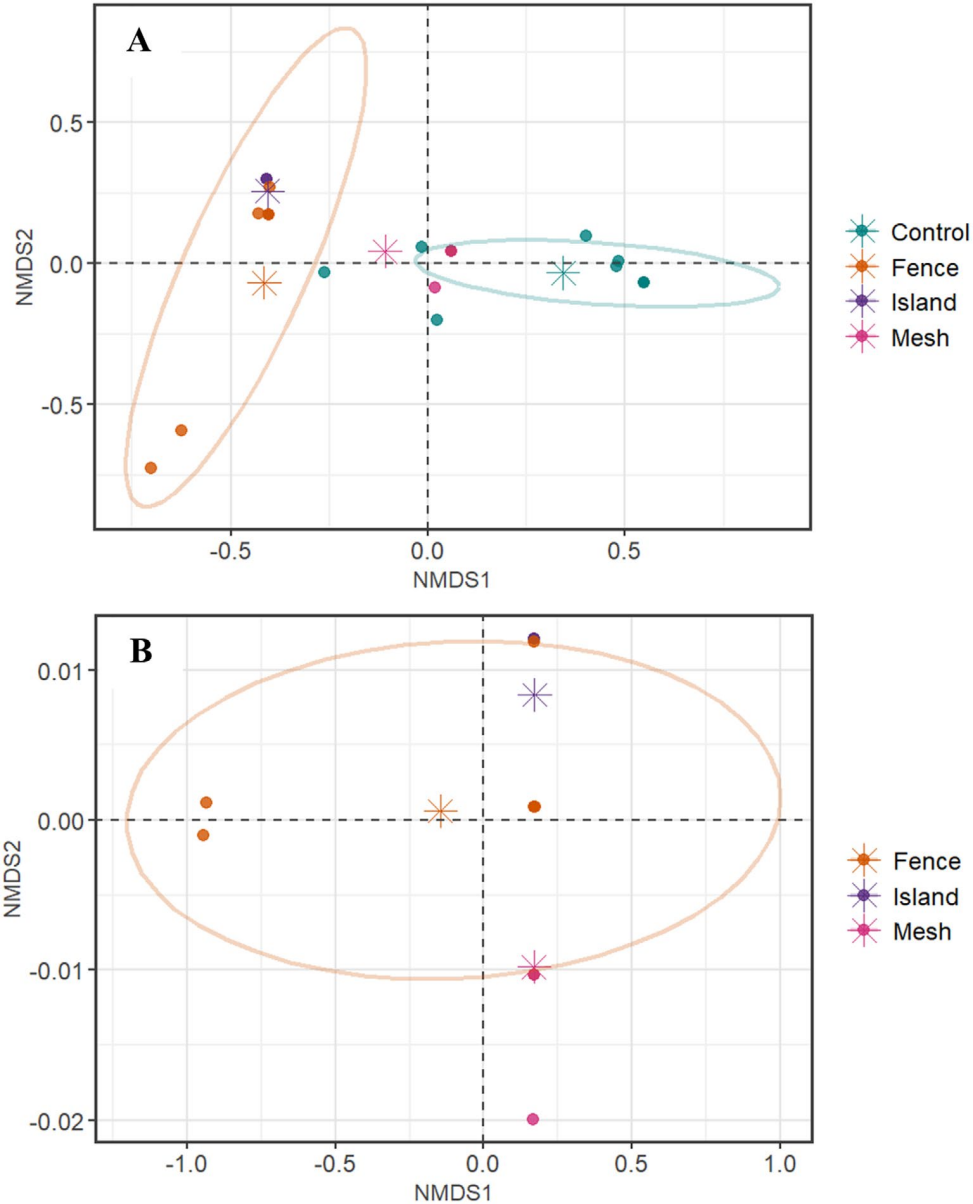
Non‐metric multidimensional scaling (NMDS) plots of predator species across different treatment groups, analyzing predation on artificial freshwater turtle nests in Victoria and South Australia, Australia during 2023 to 2024. Plot A (top) includes the control group and plot B (below) excludes the control group. The plot demonstrates the distribution of predator species based on their Euclidean distances in a multidimensional space (NMDS1 and NMDS2 axes). Each point represents a sample, colored by treatment group, with centroids (mean position of samples) indicated by asterisks. Outlined 85% confidence ellipses illustrate the dispersion of samples within each group. Ellipses could not be created for the island and mesh groups due to insufficient sample sizes.

Foxes were the dominant predator across all control plots and were responsible for 93% of all destroyed nests in this group (Table 2). Fox predation was also high in the meshed plots, comprising 83% of the total destroyed nests, while raven ( *Corvus coronoides* ) predation accounted for 69% of the nests destroyed at fenced sites. Only 17% of nests were damaged on islands, and these were entirely attributable to Australasian swamphens (*Porphyrio melanotus*). NMDS plots of predator species display all three protection treatment centroids (average of the sample, indicated by asterisks) clustered in the top and bottom left quadrants, while the control centroid is placed to the right of the central intersection (Figure 4A). Predator species of the control plots differed significantly from those seen in each protection treatment group; however, there were no significant differences in nest predator species between each of the protection treatment groups (PERMANOVA, Table 3). This difference is primarily because foxes were the main predators in the control nests, but were substantially reduced by all the nest protection methods.

**TABLE 2:** Number of destroyed artificial freshwater turtle nests and their associated predators in Victoria and South Australia, Australia 2023–2024.

Treatment	Total nests	Number of destroyed nests by predator					Total destroyed nests
		Fox	Raven	Swamphen	Possum	Echidna	
Control	115	94	4	2	—	1	101
Fence	70	5	18	2	1	—	26
Mesh	30	10	2	—	—	—	12
Island	12	—	—	2	—	—	2

**TABLE 3:** PERMANOVA comparison of predator species across treatments for artificial freshwater turtle nest predation in Victoria and South Australia, 2023–2024.

Treatment	Df	*R* ^2^	*F*	*p*
Control vs. Fence	3, 22	0.50	17.90	< 0.001*
Control vs. Island	3, 22	0.57	18.90	0.003*
Control vs. Mesh	3, 22	0.28	5.35	0.026*
Fence vs. Island	3, 22	0.11	1.03	0.276
Fence vs. Mesh	3, 22	0.12	1.09	0.326
Island vs. Mesh	3, 22	0.48	3.66	0.200

*Note:* Asterisks (*) indicate significant pairwise differences.

As the control group was dominated by foxes as the primary predators, the NMDS analysis was run again with the control group excluded to determine whether any differences in nest predators occurred only within protection treatments. After excluding the control group and plotting the NMDS only for nest protection treatments (Figure 4B), the mesh and island centroids are both placed within the 85% confidence ellipsis of the fence group, illustrating no significant difference from one another (Table 3). Both island and mesh groups shared predators with the fence group, but not with each other (Table 2). Thus, there was no evidence that the nest protection method substantially affected the diversity of predators capable of attacking the nests, once foxes were excluded. Overall, our analyses indicated that while native predators did attack nests in protected plots, their contribution to nest predation was considerably lower than that of foxes in unprotected control plots.

## 5 Discussion

Mesh screening, exclusion fencing, and artificial floating islands all effectively reduced fox predation on artificial nests, demonstrating that using any of these methods provides greater protection than leaving nests unprotected. We demonstrate here, for the first time, that artificial floating islands offer a uniquely secure nesting site. The islands provided the only protection strategy that foxes did not breach. Foxes destroyed 90%–100% of nests in control plots, which aligns with previous observations of similarly high predation rates of unprotected nests (Congdon et al. 1987; Munscher et al. 2012; Purger et al. 2023; Terry et al. 2023; Thompson 1983b). Once excluded, however, we were able to test whether native predators replaced the predation pressure of foxes in the presence of our exclusion methods. Determining historic native predation rates before the introduction of foxes is challenging due to a lack of historical data, and it is conceivable that when foxes are excluded native predators will destroy nests with similar frequency (Chessman 2021). In our study, native predators did raid nests following the exclusion of foxes; however, predation rates were significantly lower in protected plots than in control plots. If native predators were to directly replace foxes, then we would expect consistent predation rates across all groups. In particular, birds are easily able to access the floating islands and fenced nesting beaches and so will not be excluded by those nest protection methods. Here, the lower predation rates in protected plots suggest that native predators are not as impactful as foxes in terms of nest predation when foxes are no longer present, and that the protection methods may also reduce predation by native predators. Future research should aim to evaluate these nest protection methods over multiple nesting seasons to assess long‐term effectiveness, particularly focusing on potential compensatory increases in native predator activity when foxes are excluded.

### 5.1 Artificial Floating Islands

Artificial floating islands were the best‐performing protection method, but their effectiveness may depend on their location and the associated predator communities and abundances. While no predation occurred at one location, two nests on islands at the second site were exposed at the conclusion of the study, with camera footage indicating they were unearthed by native Australasian swamphens ( *P. melanotus* ). These birds had previously destroyed nests in other locations, indicating they will likely continue to pose a threat to nest survival on all islands. Importantly, no foxes reached any floating islands, likely reflecting an aversion to swimming or naїvety to islands more generally. Although foxes are capable of swimming to access food sources or escape danger (Angerbjörn 1989; Murie 1959), the distance required to access floating islands may serve as a sufficient deterrent for foxes, particularly if food is readily available on the shore. Given their excellent sense of smell and visual acuity (Lai et al. 2015; Petrov et al. 2018), it is possible that foxes are capable of smelling nests or spotting nesting females on floating islands within several meters of the shore, and therefore, islands should be anchored well away from the shore where possible. Floating islands can be costly to construct (typically ranging from AU$5000–$10,000) but require little ongoing maintenance (aside from the annual preparation of nesting boxes) once deployed.

Nest‐site fidelity, whereby females return to previously used nesting areas, may delay or prevent nesting on artificial islands in species with strong preferences for specific locations or microhabitat features such as soil type and vegetation cover (Janzen and Morjan 2001; Spencer and Thompson 2003; Valenzuela and Janzen 2001). Mimicking preferred on‐shore conditions on artificial islands may increase their suitability and encourage uptake by nesting females (Wilson 1998). Although no nesting on artificial islands occurred during this study (2023–24), the following season (2024–25) saw high *E. macquarii* nesting activity on one island only one month after it was launched (Hunter et al., *in preparation*), and *C. longicollis* nesting on another island within days of its launch (R. Spencer, *unpublished observation*), indicating their potential for adoption within a relatively short timeframe. For successful incubation on floating islands, the soil composition and drainage capacity of nesting boxes should be tailored around the optimum temperature and moisture conditions of the target turtle species (Wilson 1998). If incubation requirements are not known, these data should be gathered first to avoid unnecessary egg mortality through failed incubation.

### 5.2 Fence

**FIGURE 5:**
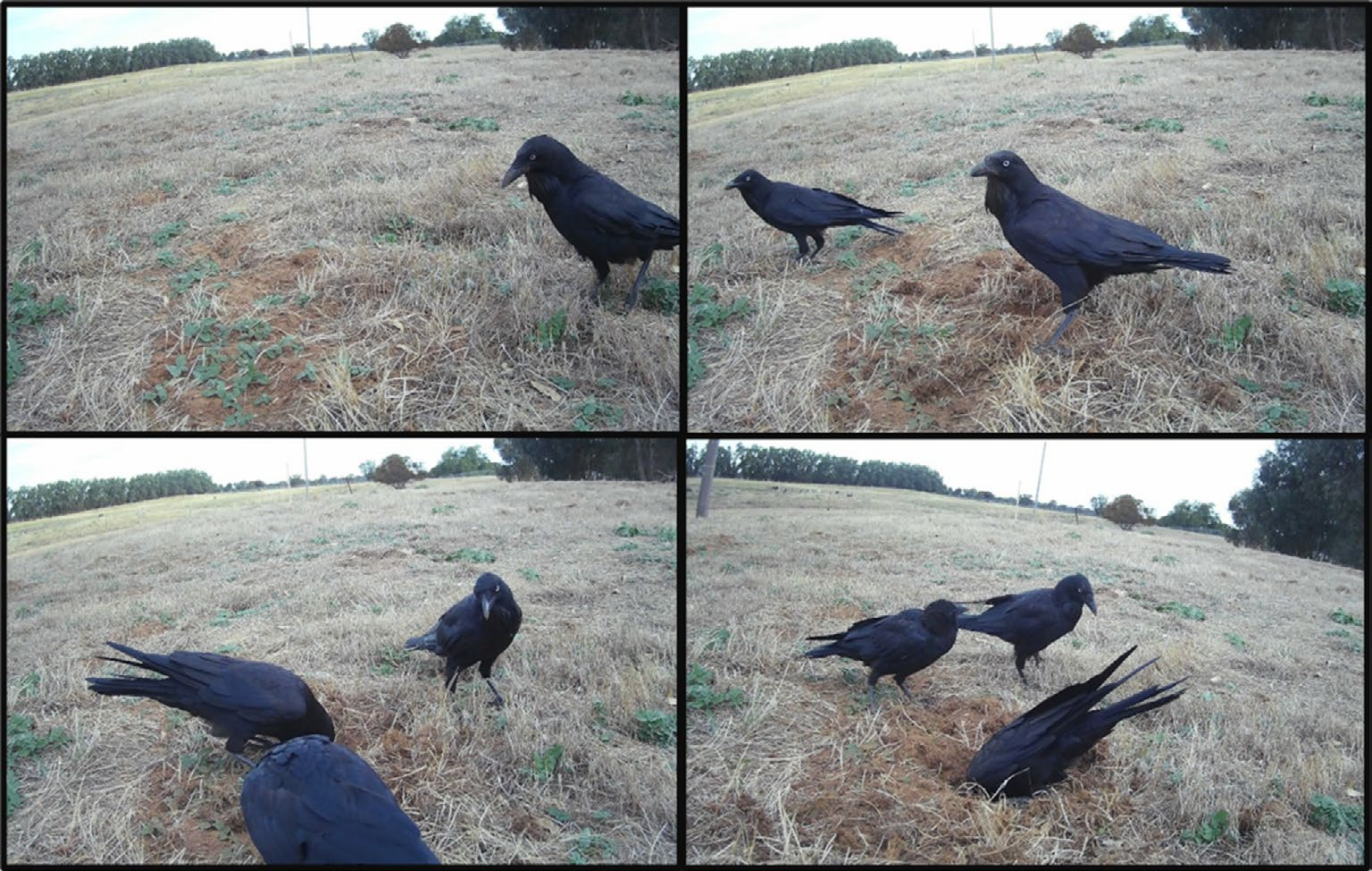
Example of raven predation at a fenced site in Victoria, Australia (2023–2024), over 15 min.

Fox predation was minimal across our fenced sites, supporting the use of fences as an effective barrier against foxes (Guyot and Kuchling 1998; Streeting et al. 2023). Native predation was also generally low; however, this varied across sites. Ravens ( *C. coronoides* ) were particularly active nest predators at one site, destroying 90% of all nests inside the fence. Ravens are predators of a broad range of turtle eggs and hatchlings (Boarman 2003; Ercolano 2008; Thompson 1983b), and typically destroyed nests in our study in groups of three animals (Figure 5). Social learning through observation of conspecifics is well recorded in corvids (Bugnyar and Kotrschal 2002; Emery and Clayton 2004; Midford et al. 2000) and this could, in part, explain the rapid group‐raiding of nests at this site. The absence of raven predation at other fenced sites might stem from an unfamiliarity with turtle eggs as a food source. Once discovered, this may lead to a significant increase in nest predation, as seen in feral pigs (*Sus scofra*; Engeman et al. 2016).

Unlike in open systems, eradication of pest predators is feasible inside fenced areas (Legge et al. 2018), and before creating artificial nest plots there were no fox dens identified within any fenced sites used in our study. However, a fox did enter a fenced site on one occasion when lowering water levels caused by a short drought likely allowed it to walk around the edge of the fence and destroy 50% of nests inside. In the future, fox incursions could be avoided by extending the end posts of the fence far enough into the water to ensure the area remains enclosed even during dry periods (Streeting et al. 2023). Even with this deterrent, however, foxes may swim in around the outskirts of fenced sites (Dickman 2011).

Similar to floating islands, fenced nesting sites require little ongoing labour aside from occasional maintenance. However, their construction can entail reasonably high upfront costs and may disturb or alter nesting habitat, potentially affecting the likelihood of turtles nesting there. While we did not observe any turtles trapped in the fences used in our study, fencing may pose an entrapment risk to dispersing females (Ferronato et al. 2014), as well as other wildlife, and require alterations; see (Dowling et al. 2024; Guyot and Kuchling 1998; Waltham et al. 2022). Further research on the value of fenced nesting sites in relation to real turtle nesting behavior would benefit future management decisions.

### 5.3 Mesh

**FIGURE 6:**
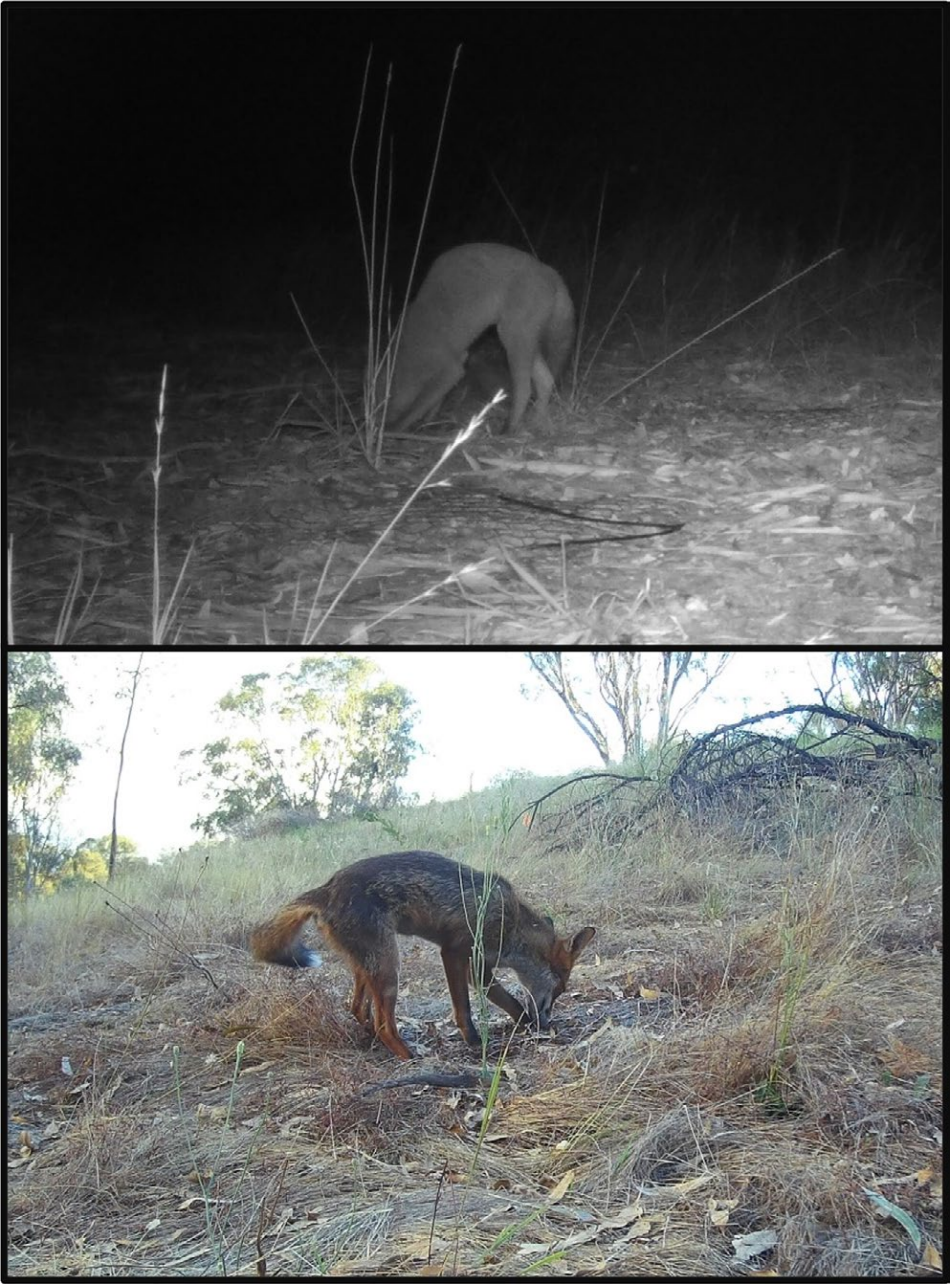
Example of foxes using their snouts (A) and paws (B) to access chicken eggs in mesh‐covered nests in Victoria, Australia 2023–2024.

While better than no protection at all, mesh was the poorest‐performing protection treatment, and experienced average predation rates (40%) similar to those seen in other studies using mesh to deter turtle nest predators (Nordberg et al. 2019; O'Connor et al. 2017; Terry 2024). Foxes can break into nests by pushing their snouts or paws through the holes in the wire (Figure 6), and while a finer aperture wire may prevent intrusion, the gap would not allow for hatchling turtles to pass through when emerging from the nest. The risk of predators breaking through the mesh is most likely determined by the type of material used in relation to the types of predators being controlled for (Lovemore et al. 2020; Pheasey et al. 2018). For instance, plastic mesh may be sufficient to protect nests from dingoes ( *Canis familiaris* ; Nordberg et al. 2019), monitor lizards ( *Varanus panoptes* ; Lei and Booth 2017) and coyotes ( *Canis latrans* ; Lovemore et al. 2020), but may be less effective against foxes. As an example, foxes easily chew through even multiple layers of plastic mesh resulting in up to 100% depredation of turtle nests (Terry et al. 2023). Similarly, wire cages can afford sufficient protection from foxes and raccoons ( *Procyon lotor* ; Kurz et al. 2012; Streeting et al. 2023), but not feral pigs (*S. scofra*; Engeman et al. 2016).

For predators that primarily hunt using olfactory cues, such as foxes, it is unlikely that the visual presence of protective mesh alone increases predation rates over time through an association with food (Bowen and Janzen 2005; Jobe et al. 2023; Terry 2024). However, other predators using a combination of visual, tactile and olfactory cues may learn to associate mesh with food, thereby focusing their predation efforts on these markers (Bougie et al. 2020; Mroziak et al. 2000; Rollinson and Brooks 2007; Schindler et al. 2017; Williams et al. 2020). Although our study did not incorporate these techniques, covering mesh with a fine layer of sand or soil may reduce predator detection of protected nests (Kurz et al. 2012; O'Connor et al. 2017). While mesh may not be a visual cue for foxes, its use will likely have diminishing returns if breakthroughs are left unchecked and concerted efforts by foxes consistently result in a food reward (Niehaus et al. 2004). Quickly replacing broken mesh (or other exclusion devices) with sturdier alternatives can modify fox behavior, leading them to ignore protected nests once they learn they cannot break through (O'Connor et al. 2017). In corvids, 85% of predation events ended within two minutes when birds failed to access nests through exclusion cages (Major et al. 2015).

A final limitation in using mesh as a protection method is the need to find intact turtle nests, which can be highly cryptic (Ratnaswamy et al. 1997; Terry et al. 2023; Wirsing et al. 2012). The in‐person hours required to find nests can be expensive if this is paid time (Ratnaswamy et al. 1997), and subsequently, many projects rely on volunteers (O'Connor et al. 2017; Riley and Litzgus 2013), or additional cues such as trail cameras or scent detection dogs (Streeting et al. 2023).

### 5.4 Management Implications

While artificial floating islands are a novel conservation method for freshwater turtles, our findings provide encouraging evidence supporting their continued use and further research into their potential benefits and optimized design. We note, however, that these findings are preliminary and limited by the small sample sizes within the island treatment and therefore should be interpreted as indicative rather than conclusive. Both artificial floating islands and exclusion fencing provide scalable, long‐term nest protection solutions but require significant initial investments and resource commitments. Mesh protection is labor‐intensive due to the effort required to locate nests, but it is likely a more cost‐effective option for protecting individual nests because the materials required are inexpensive (less than $1 AUD per nest). In high‐risk areas, mesh may require reinforcement with exclusion cages or rigid wire to effectively prevent predator access. A potential limitation in using chicken eggs is that differences in odor compared to freshly laid turtle eggs could underestimate predation rates. However, as our objective was to compare the *relative* effectiveness of the protection methods rather than measure absolute predation rates, we believe this limitation does not significantly compromise our conclusions. In conjunction with monitoring nest predation rates, further monitoring of nesting preferences in female turtles and the survival of resulting hatchlings is needed to accurately assess the cost–benefit and practical effectiveness of floating islands, fences, and mesh as conservation strategies.

## Author Contributions

**Christina Hunter:** conceptualization (equal), formal analysis (lead), investigation (lead), methodology (equal), writing – original draft (lead). **Deborah S. Bower:** conceptualization (equal), formal analysis (supporting), funding acquisition (equal), investigation (supporting), supervision (supporting), writing – review and editing (supporting). **Richard A. Peters:** formal analysis (supporting), investigation (supporting), supervision (supporting), writing – review and editing (supporting). **Ricky‐John Spencer:** conceptualization (equal), funding acquisition (supporting), investigation (supporting), writing – review and editing (supporting). **Ligia Pizzatto:** data curation (supporting), investigation (supporting), project administration (supporting), writing – review and editing (supporting). **James U. Van Dyke:** conceptualization (lead), data curation (lead), formal analysis (supporting), funding acquisition (lead), investigation (supporting), methodology (supporting), project administration (lead), supervision (lead), writing – review and editing (equal).

## Conflicts of Interest

The authors declare no conflicts of interest.

## Supporting information


**Data S1:** ece372121‐sup‐0001‐DataS1.xlsx.

**Data S2:** ece372121‐sup‐0002‐Supinfo.docx.

## Data Availability

All data from this manuscript are included as Supporting Information.

## References

[ece372121-bib-0001] Angerbjörn, A. 1989. “Mountain Hare Populations on Islands: Effects of Predation by Red Fox.” Oecologia 81: 335–340. 28311185 10.1007/BF00377080

[ece372121-bib-0002] Baggiano, O. 2012. The Murray–Darling Turtles: Gene Flow and Population Persistence in Dryland Rivers. In: Doctor of Philosophy, Griffith University, Nathan, Australia.

[ece372121-bib-0003] Banks, P. B. , and C. R. Dickman . 2007. “Alien Predation and the Effects of Multiple Levels of Prey Naiveté.” Trends in Ecology & Evolution 22, no. 5: 229–230. 17300855 10.1016/j.tree.2007.02.006

[ece372121-bib-0004] Boarman, W. I. 2003. “Managing a Subsidized Predator Population: Reducing Common Raven Predation on Desert Tortoises.” Environmental Management 32, no. 2: 205–217. 14753646 10.1007/s00267-003-2982-x

[ece372121-bib-0005] Booth, D. T. 2003. “Composition and Energy Density of Eggs From Two Species of Freshwater Turtle With Twofold Ranges in Egg Size.” Comparative Biochemistry and Physiology Part A: Molecular & Integrative Physiology 134, no. 1: 129–137. 10.1016/s1095-6433(02)00216-7 12507616

[ece372121-bib-0006] Bougie, T. , N. Byer , C. Lapin , M. Z. Peery , J. E. Woodford , and J. N. Pauli . 2020. “Wood Turtle (*Glyptemys insculpta*) Nest Protection Reduces Depredation and Increases Success, but Annual Variation Influences Its Effectiveness.” Canadian Journal of Zoology 98, no. 11: 715–724.

[ece372121-bib-0007] Bowen, K. D. , and F. J. Janzen . 2005. “Rainfall and Depredation of Nests of the Painted Turtle, *Chrysemys picta* .” Journal of Herpetology 39, no. 4: 649–652.

[ece372121-bib-0008] Brooks, M. E. , K. Kristensen , K. J. Van Benthem , et al. 2017. “glmmTMB Balances Speed and Flexibility Among Packages for Zero‐Inflated Generalized Linear Mixed Modeling.” R Journal 9, no. 2: 378–400.

[ece372121-bib-0009] Bugnyar, T. , and K. Kotrschal . 2002. “Observational Learning and the Raiding of Food Caches in Ravens, *Corvus Corax*: Is It ‘Tactical’ Deception?” Animal Behaviour 64, no. 2: 185–195.

[ece372121-bib-0010] Campbell, M. A. , M. J. Connell , S. J. Collett , et al. 2020. “The Efficacy of Protecting Turtle Nests as a Conservation Strategy to Reverse Population Decline.” Biological Conservation 251: 108769.

[ece372121-bib-0011] Chessman, B. C. 2011. “Declines of Freshwater Turtles Associated With Climatic Drying in Australia's Murray–Darling Basin.” Wildlife Research 38, no. 8: 664–671.

[ece372121-bib-0012] Chessman, B. C. 2021. “Introduced Red Foxes (*Vulpes vulpes*) Driving Australian Freshwater Turtles to Extinction? A Critical Evaluation of the Evidence.” Pacific Conservation Biology 28, no. 6: 462–471.

[ece372121-bib-0013] Christiansen, J. , and B. Gallaway . 1984. “Raccoon Removal, Nesting Success, and Hatchling Emergence in Iowa Turtles With Special Reference to *Kinosternon flavescens* (Kinosternidae).” Southwestern Naturalist 29: 343–348.

[ece372121-bib-0014] Congdon, J. D. , G. L. Breitenbach , R. C. van Loben Sels , and D. W. Tinkle . 1987. “Reproduction and Nesting Ecology of Snapping Turtles (*Chelydra serpentina*) in Southeastern Michigan.” Herpetologica 43: 39–54.

[ece372121-bib-0015] Congdon, J. D. , A. E. Dunham , and R. van Loben Sels . 1993. “Delayed Sexual Maturity and Demographics of Blanding's Turtles (*Emydoidea blandingii*): Implications for Conservation and Management of Long‐Lived Organisms.” Conservation Biology 7, no. 4: 826–833.

[ece372121-bib-0016] Congdon, J. D. , D. W. Tinkle , G. L. Breitenbach , and R. C. van Loben Sels . 1983. “Nesting Ecology and Hatching Success in the Turtle *Emydoidea blandingi* .” Herpetologica 39: 417–429.

[ece372121-bib-0017] Dawson, S. J. , P. J. Adams , R. M. Huston , and P. A. Fleming . 2014. “Environmental Factors Influence Nest Excavation by Foxes.” Journal of Zoology 294, no. 2: 104–113.

[ece372121-bib-0018] Dickman, C. R. 2011. “Fences or Ferals? Benefits and Costs of Conservation Fencing in Australia.” In Fencing for Conservation: Restriction of Evolutionary Potential or a Riposte to Threatening Processes?, 43–63. Springer.

[ece372121-bib-0019] Doherty, T. S. , C. R. Dickman , D. G. Nimmo , and E. G. Ritchie . 2015. “Multiple Threats, or Multiplying the Threats? Interactions Between Invasive Predators and Other Ecological Disturbances.” Biological Conservation 190: 60–68.

[ece372121-bib-0020] Doherty, T. S. , A. S. Glen , D. G. Nimmo , E. G. Ritchie , and C. R. Dickman . 2016. “Invasive Predators and Global Biodiversity Loss.” Proceedings of the National Academy of Sciences 113, no. 40: 11261–11265. 10.1073/pnas.1602480113 PMC5056110 27638204

[ece372121-bib-0021] Doherty, T. S. , and E. G. Ritchie . 2017. “Stop Jumping the Gun: A Call for Evidence‐Based Invasive Predator Management.” Conservation Letters 10, no. 1: 15–22.

[ece372121-bib-0022] Dowling, J. M. , D. S. Bower , R. Boscarino‐Gaetano , and E. J. Nordberg . 2024. “The Influence of Fence Design on the Movement Patterns of Eastern Long‐Necked Turtles.” Journal of Wildlife Management 88: e22654.

[ece372121-bib-0023] Emery, N. J. , and N. S. Clayton . 2004. “The Mentality of Crows: Convergent Evolution of Intelligence in Corvids and Apes.” Science 306, no. 5703: 1903–1907. 15591194 10.1126/science.1098410

[ece372121-bib-0024] Engeman, R. M. , D. Addison , and J. Griffin . 2016. “Defending Against Disparate Marine Turtle Nest Predators: Nesting Success Benefits From Eradicating Invasive Feral Swine and Caging Nests From Raccoons.” Oryx 50, no. 2: 289–295.

[ece372121-bib-0025] Ercolano, E. 2008. “Aquatic and Terrestrial Habitat Use of the Australian Freshwater Turtle, *Chelodina expansa*.” Independent Study Project (ISP) Collection. SIT Study Abroads. https://digitalcollections.sit.edu/isp_collection/56/.

[ece372121-bib-0026] Fea, N. , W. Linklater , and S. Hartley . 2021. “Responses of New Zealand Forest Birds to Management of Introduced Mammals.” Conservation Biology 35, no. 1: 35–49. 31893568 10.1111/cobi.13456 PMC7984369

[ece372121-bib-0027] Ferronato, B. O. , J. H. Roe , and A. Georges . 2014. “Reptile Bycatch in a Pest‐Exclusion Fence Established for Wildlife Reintroductions.” Journal for Nature Conservation 22, no. 6: 577–585.

[ece372121-bib-0028] Fulton, G. R. , and H. A. Ford . 2001. “The Pied Currawong's Role in Avian Nest Predation: A Predator Removal Experiment.” Pacific Conservation Biology 7, no. 3: 154–160.

[ece372121-bib-0029] Gautschi, D. , A. Čulina , R. Heinsohn , D. Stojanovic , and R. Crates . 2024. “Protecting Wild Bird Nests Against Predators: A Systematic Review and Meta‐Analysis of Non‐Lethal Methods.” Journal of Applied Ecology 61, no. 6: 1187–1198.

[ece372121-bib-0030] Geller, G. A. 2012. “Reducing Predation of Freshwater Turtle Nests With a Simple Electric Fence.” Herpetological Review 43, no. 3: 398.

[ece372121-bib-0031] Georges, A. , and R. Kennett . 1989. “Dry‐Season Distribution and Ecology of *Carettochelys insculpta* (Chelonia: Carettochelydidae) in Kakadu National Park, Northern Australia.” Australian Wildlife Research 16: 323–335.

[ece372121-bib-0095] Grosse, A. M. , B. A. Crawford , J. C. Maerz , et al. 2015. “Effects of Vegetation Structure and Artificial Nesting Habitats on Hatchling Sex Determination and Nest Survival of Diamondback Terrapins.” Journal of Fish and Wildlife Management 6, no. 1: 19–28. 10.3996/082014-jfwm-063.

[ece372121-bib-0032] Guyot, G. , and G. Kuchling . 1998. “One Way‐Gates for a Fenced Population of the Australian Western Swamp Turtle, *Pseudemydura umbrina* .” In Proceedings of the 3rd International Symposium on Emys Orbicularis and Other European Freshwater Turtles, 167–172. Societas Europaea Herpetologica, Le Bourget du Lac.

[ece372121-bib-0033] Hancock, M. 2000. “Artificial Floating Islands for Nesting Black‐Throated Divers *Gavia Arctica* in Scotland: Construction, Use and Effect on Breeding Success.” Bird Study 47, no. 2: 165–175.

[ece372121-bib-0034] Holling, C. S. 1959. “The Components of Predation as Revealed by a Study of Small‐Mammal Predation of the European Pine Sawfly.” Canadian Entomologist 91, no. 5: 293–320.

[ece372121-bib-0035] Janzen, F. J. , and C. L. Morjan . 2001. “Repeatability of Microenvironment‐Specific Nesting Behaviour in a Turtle With Environmental Sex Determination.” Animal Behaviour 62, no. 1: 73–82.

[ece372121-bib-0036] Jobe, S. , R. E. Urbanek , P. Hillbrand , E. S. Darrow , and E. Abernethy . 2023. “Predator Exclusion Cages as Visual Attractants to Coyotes.” Urban Ecosystems 26, no. 4: 981–989.

[ece372121-bib-0037] Karstens, S. , M. Langer , H. Nyunoya , et al. 2021. “Constructed Floating Wetlands Made of Natural Materials as Habitats in Eutrophicated Coastal Lagoons in the Southern Baltic Sea.” Journal of Coastal Conservation 25, no. 4: 44.

[ece372121-bib-0038] Kennett, R. , K. E. Hodges , and A. Georges . 2009. *Chelodina longicollis* (Shaw 1794)–Eastern Long‐Necked Turtle, Common Long‐Necked Turtle (Conservation Biology of Freshwater Turtles and Tortoises: A Compilation Project of the IUCNSSC Tortoise and Freshwater Turtle Specialist Group, Issue). C. R Foundation.

[ece372121-bib-0039] Kurz, D. J. , K. M. Straley , and B. A. DeGregorio . 2012. “Out‐Foxing the Red Fox: How Best to Protect the Nests of the Endangered Loggerhead Marine Turtle *Caretta caretta* From Mammalian Predation?” Oryx 46, no. 2: 223–228.

[ece372121-bib-0040] Lai, S. , J. Bêty , and D. Berteaux . 2015. “Spatio–Temporal Hotspots of Satellite–Tracked Arctic Foxes Reveal a Large Detection Range in a Mammalian Predator.” Movement Ecology 3: 1–10. 26568827 10.1186/s40462-015-0065-2 PMC4644628

[ece372121-bib-0041] Legge, S. , J. C. Woinarski , A. A. Burbidge , et al. 2018. “Havens for Threatened Australian Mammals: The Contributions of Fenced Areas and Offshore Islands to the Protection of Mammal Species Susceptible to Introduced Predators.” Wildlife Research 45, no. 7: 627–644.

[ece372121-bib-0042] Lei, J. , and D. T. Booth . 2017. “How Best to Protect the Nests of the Endangered Loggerhead Turtle *Caretta caretta* From Monitor Lizard Predation.” Chelonian Conservation and Biology 16, no. 2: 246–249.

[ece372121-bib-0043] Lenth, R. V. 2024. “emmeans: Estimated Marginal Means, aka Least‐Squares Means.” https://rvlenth.github.io/emmeans/, https://rvlenth.github.io/emmeans/.

[ece372121-bib-0044] Lovemore, T. E. , N. Montero , S. A. Ceriani , and M. M. Fuentes . 2020. “Assessing the Effectiveness of Different Sea Turtle Nest Protection Strategies Against Coyotes.” Journal of Experimental Marine Biology and Ecology 533: 151470.

[ece372121-bib-0045] Lovich, J. E. , J. R. Ennen , M. Agha , and J. W. Gibbons . 2018. “Where Have All the Turtles Gone, and Why Does It Matter?” Bioscience 68, no. 10: 771–781. 10.1093/biosci/biy095.

[ece372121-bib-0046] Major, R. E. , M. B. Ashcroft , and A. Davis . 2015. “Nest Caging as a Conservation Tool for Threatened Songbirds.” Wildlife Research 41, no. 7: 598–605.

[ece372121-bib-0047] Manikowska‐Ślepowrońska, B. , K. Ślepowroński , and D. Jakubas . 2022. “The Use of Artificial Floating Nest Platforms as Conservation Measure for the Common Tern *Sterna hirundo*: A Case Study in the RAMSAR Site Druzno Lake in Northern Poland.” European Zoological Journal 89, no. 1: 229–240.

[ece372121-bib-0048] McIntyre, J. W. , and J. E. Mathisen . 1977. “Artificial Islands as Nest Sites for Common Loons.” Journal of Wildlife Management 41, no. 2: 317–319.

[ece372121-bib-0049] Menezes, J. C. , and M. Â. Marini . 2017. “Predators of Bird Nests in the Neotropics: A Review.” Journal of Field Ornithology 88, no. 2: 99–114.

[ece372121-bib-0050] Midford, P. E. , J. P. Hailman , and G. E. Woolfenden . 2000. “Social Learning of a Novel Foraging Patch in Families of Free‐Living Florida Scrub‐Jays.” Animal Behaviour 59, no. 6: 1199–1207. 10877899 10.1006/anbe.1999.1419

[ece372121-bib-0051] Mroziak, M. L. , M. Salmon , and K. Rusenko . 2000. “Do Wire Cages Protect Sea Turtles From Foot Traffic and Mammalian Predators?” Chelonian Conservation and Biology 3, no. 4: 693–698.

[ece372121-bib-0052] Mullin, D. I. , R. C. White , J. L. Mullen , A. M. Lentini , R. J. Brooks , and J. D. Litzgus . 2023. “Headstarting Turtles to Larger Body Sizes for Multiple Years Increases Survivorship but With Diminishing Returns.” Journal of Wildlife Management 87, no. 4: e22390. 10.1002/jwmg.22390.

[ece372121-bib-0053] Munscher, E. C. , E. H. Kuhns , C. A. Cox , and J. A. Butler . 2012. “Decreased Nest Mortality for the Carolina Diamondback Terrapin (*Malaclemys Terrapin Centrata*) Following Removal of Raccoons (*Procyon lotor*) From a Nesting Beach in Northeastern Florida.” Herpetological Conservation and Biology 7, no. 2: 176–184.

[ece372121-bib-0054] Murie, O. J. 1959. Fauna of the Aleutian Islands and Alaska Peninsula. Department of the Interior, US Fish and Wildlife Service.

[ece372121-bib-0055] Nakamura, K. , and G. Mueller . 2008. Review of the Performance of the Artificial Floating Island as a Restoration Tool for Aquatic Environments. World Environmental and Water Resources Congress 2008: Ahupua'A.

[ece372121-bib-0056] Niehaus, A. C. , D. R. Ruthrauff , and B. J. McCaffery . 2004. “Response of Predators to Western Sandpiper Nest Exclosures.” Waterbirds 27, no. 1: 79–82.

[ece372121-bib-0057] Nordberg, E. J. , S. Macdonald , G. Zimny , et al. 2019. “An Evaluation of Nest Predator Impacts and the Efficacy of Plastic Meshing on Marine Turtle Nests on the Western Cape York Peninsula, Australia.” Biological Conservation 238: 108201. 10.1016/j.biocon.2019.108201.

[ece372121-bib-0058] O'Connor, J. M. , C. J. Limpus , K. M. Hofmeister , B. L. Allen , and S. E. Burnett . 2017. “Anti‐Predator Meshing May Provide Greater Protection for Sea Turtle Nests Than Predator Removal.” PLoS One 12, no. 2: e0171831. 28187181 10.1371/journal.pone.0171831 PMC5302370

[ece372121-bib-0059] Oksanen, J. , G. Simpson , F. Blanchet , et al. 2023. vegan: Community Ecology Package. R package version 2.6–4.

[ece372121-bib-0060] Petrov, K. , H. Stricker , J. U. Van Dyke , G. Stockfeld , P. West , and R.‐J. Spencer . 2018. “Nesting Habitat of the Broad‐Shelled Turtle (*Chelodina expansa*).” Australian Journal of Zoology 66, no. 1: 4–14.

[ece372121-bib-0061] Pheasey, H. , M. McCargar , A. Glinsky , and N. Humphreys . 2018. “Effectiveness of Concealed Nest Protection Screens Against Domestic Predators for Green (*Chelonia mydas*) and Hawksbill (*Eretmochelys imbricata*) Sea Turtles.” Chelonian Conservation and Biology 17, no. 2: 263–270.

[ece372121-bib-0062] Purger, J. J. , T. G. Molnár , Z. Lanszki , and J. Lanszki . 2023. “European Pond Turtle (*Emys orbicularis*) Nest Predation: A Study With Artificial Nests.” Biology 12, no. 3: 342. 36979034 10.3390/biology12030342 PMC10045932

[ece372121-bib-0063] Ratnaswamy, M. J. , R. J. Warren , M. T. Kramer , and M. D. Adam . 1997. “Comparisons of Lethal and Nonlethal Techniques to Reduce Raccoon Depredation of Sea Turtle Nests.” Journal of Wildlife Management 61: 368–376.

[ece372121-bib-0064] Ricklefs, R. E. 1969. “An Analysis of Nesting Mortality in Birds.” Auk 87.4: 826–828.

[ece372121-bib-0065] Riley, J. L. , and J. D. Litzgus . 2013. “Evaluation of Predator‐Exclusion Cages Used in Turtle Conservation: Cost Analysis and Effects on Nest Environment and Proxies of Hatchling Fitness.” Wildlife Research 40, no. 6: 499. 10.1071/wr13090.

[ece372121-bib-0066] Robinson, K. J. , D. J. Limpus , B. Crosbie , C. J. Limpus , and L. D. Fabbro . 2024. “Depredation of Eggs of Threatened Freshwater Turtles by the Short‐Beaked Echidna ( *Tachyglossus aculeatus* (Shaw, 1792)).” Australian Journal of Zoology 71, no. 4: ZO23029.

[ece372121-bib-0067] Robley, A. , L. Woodford , M. Thompson , A. Taglierini , and B. Hradsky . 2018. “Protecting Hattah–Kulkyne Ramsar Wetlands From Introduced Predators: Final Report 2017–2018.” Arthur Rylah Institute for Environmental Research Technical Report Series.

[ece372121-bib-0068] Rollinson, N. , and R. J. Brooks . 2007. “Marking Nests Increases the Frequency of Nest Depredation in a Northern Population of Painted Turtles ( *Chrysemys picta* ).” Journal of Herpetology 41, no. 1: 174–176.

[ece372121-bib-0069] Saunders, G. R. , M. N. Gentle , and C. R. Dickman . 2010. “The Impacts and Management of Foxes *Vulpes vulpes* in Australia.” Mammal Review 40, no. 3: 181–211.

[ece372121-bib-0070] Schindler, M. , H. Frötscher , A. Hille , M. R. Bruck , M. Schmidt , and Y. V. Kornilev . 2017. “Nest Protection During a Long‐Term Conservation Project as a Tool to Increase the Autochthonous Population of *Emys orbicularis* (L., 1758) in Austria.” Acta Zoologica Bulgarica 2017: 147–154.

[ece372121-bib-0071] Schwanz, L. E. , R.‐J. Spencer , R. M. Bowden , and F. J. Janzen . 2010. “Climate and Predation Dominate Juvenile and Adult Recruitment in a Turtle With Temperature‐Dependent Sex Determination.” Ecology 91, no. 10: 3016–3026. 21058561 10.1890/09-1149.1

[ece372121-bib-0072] Smith, R. K. , A. S. Pullin , G. B. Stewart , and W. J. Sutherland . 2010. “Effectiveness of Predator Removal for Enhancing Bird Populations.” Conservation Biology 24, no. 3: 820–829. 20067492 10.1111/j.1523-1739.2009.01421.x

[ece372121-bib-0073] Solomon, M. E. 1949. “The Natural Control of Animal Populations.” Journal of Animal Ecology 18: 1–35.

[ece372121-bib-0078] Spencer, R. J. , J. U. Van Dyke , and M. B. Thompson . 2017. “Critically Evaluating Best Management Practices for Preventing Freshwater Turtle Extinctions.” Conservation Biology 31, no. 6: 1340–1349. 10.1111/cobi.12930. 28319283

[ece372121-bib-0074] Spencer, R.‐J. 2002. “Experimentally Testing Nest Site Selection: Fitness Trade‐Offs and Predation Risk in Turtles.” Ecology 83, no. 8: 2136–2144.

[ece372121-bib-0075] Spencer, R.‐J. 2018. “How Much Long‐Term Data Are Required to Effectively Manage a Wide‐Spread Freshwater Turtle?” Australian Zoologist 39, no. 4: 568–575.

[ece372121-bib-0076] Spencer, R.‐J. , and M. B. Thompson . 2003. “The Significance of Predation in Nest Site Selection of Turtles: An Experimental Consideration of Macro‐ and Microhabitat Preferences.” Oikos 102, no. 3: 592–600.

[ece372121-bib-0077] Spencer, R.‐J. , and M. B. Thompson . 2005. “Experimental Analysis of the Impact of Foxes on Freshwater Turtle Populations.” Conservation Biology 19, no. 3: 845–854. 10.1111/j.1523-1739.2005.00487.x.

[ece372121-bib-0079] Spinks, P. Q. , G. B. Pauly , J. J. Crayon , and H. B. Shaffer . 2003. “Survival of the Western Pond Turtle (*Emys marmorata*) in an Urban California Environment.” Biological Conservation 113, no. 2: 257–267.

[ece372121-bib-0080] Stantial, M. L. , J. B. Cohen , A. J. Darrah , S. L. Farrell , and B. Maslo . 2021. “The Effect of Top Predator Removal on the Distribution of a Mesocarnivore and Nest Survival of an Endangered Shorebird.” Avian Conservation and Ecology 16, no. 1: art8.

[ece372121-bib-0081] Stojanovic, D. , S. Eyles , H. Cook , F. Alves , M. Webb , and R. Heinsohn . 2019. “Photosensitive Automated Doors to Exclude Small Nocturnal Predators From Nest Boxes.” Animal Conservation 22, no. 3: 297–301.

[ece372121-bib-0082] Streeting, L. M. , M. L. Dillon , J. Nesbitt , et al. 2023. “A Shocking Result—Electric Fences Protect Western Saw‐Shelled Turtle ( *Myuchelys bellii* ) Nests From Depredation by Foxes.” Austral Ecology 48: 1571–1587.

[ece372121-bib-0083] Terry, R. 2024. Assessing Mesh for Protecting Individual Turtle Nests Against Red Fox (*Vulpes vulpes*) Predation, Master's thesis,La Trobe Univerity, Bundoora, Australia.

[ece372121-bib-0084] Terry, R. , K. A. Robert , A. Simms , G. Stockfeld , and J. U. Van Dyke . 2023. “Ineffectiveness of Plastic Mesh for Protecting Artificial Freshwater Turtle Nests From Red Fox ( *Vulpes vulpes* ) Predation.” Austral Ecology 48: 1547–1558. 10.1111/aec.13362.

[ece372121-bib-0085] Thompson, M. B. 1983a. The Physiology and Ecology of the Eggs of the Pleurodiran Tortoise *Emydura macquarii* (Gray).

[ece372121-bib-0086] Thompson, M. B. 1983b. “Populations of the Murray River Tortoise, *Emydura* (*Chelodina*): The Effect of Egg Predation by the Red Fox, *Vulpes vulpes* .” Wildlife Research 10, no. 2: 363–371.

[ece372121-bib-0087] Valenzuela, N. , and F. Janzen . 2001. Nest‐Site Philopatry and the Evolution of Temperature‐Dependent Sex Determination.

[ece372121-bib-0088] Waltham, N. J. , J. Schaffer , S. Walker , J. Perry , and E. Nordberg . 2022. “Simple Fence Modification Increases Land Movement Prospects for Freshwater Turtles on Floodplains.” Wildlife Biology 2022, no. 3: e01012.

[ece372121-bib-0089] Webb, J. K. , B. W. Brook , and R. Shine . 2002. “What Makes a Species Vulnerable to Extinction? Comparative Life‐History Traits of Two Sympatric Snakes.” Ecological Research 17: 59–67.

[ece372121-bib-0090] Wickham, H. , R. François , L. Henry , K. Müller , and D. Vaughan . 2023. “Dplyr: A Grammar of Data Manipulation.” R Package Version 1.1.2. https://dplyr.tidyverse.org.

[ece372121-bib-0091] Williams, J. , S. Pierce , M. Hamann , and M. Fuentes . 2020. “Protection of In Situ Sea Turtle Nests From Depredation.” Oceanography 11: 1–12.

[ece372121-bib-0092] Wilson, D. S. 1998. “Nest‐Site Selection: Microhabitat Variation and Its Effects on the Survival of Turtle Embryos.” Ecology 79, no. 6: 1884–1892.

[ece372121-bib-0093] Wirsing, A. J. , J. R. Phillips , M. E. Obbard , and D. L. Murray . 2012. “Incidental Nest Predation in Freshwater Turtles: Inter‐ and Intraspecific Differences in Vulnerability Are Explained by Relative Crypsis.” Oecologia 168: 977–988. 22009341 10.1007/s00442-011-2158-y

[ece372121-bib-0094] Yerli, S. , A. Canbolat , L. Brown , and D. Macdonald . 1997. “Mesh Grids Protect Loggerhead Turtle *Caretta caretta* Nests From Red Fox *Vulpes vulpes* Predation.” Biological Conservation 82, no. 1: 109–111.

