# Supplementary material for: Mitigating Fox Predation on Freshwater Turtle Nests: Comparing Effectiveness of Three In Situ Protection Methods
Source: Ecol Evol. 2025 Sep 8;15(9):e72121. doi: 10.1002/ece3.72121 (PMC12416911; doi:10.1002/ece3.72121)
Supplement: Supplementary file 2 — Data S2: ece372121‐sup‐0002‐Supinfo.docx. [file ECE3-15-e72121-s002.docx]

**Read me ⎯ data for "Mitigating fox predation on freshwater turtle nests: comparing effectiveness of three in situ protection methods"**

**Sheet 1: Artificial nest data**

This was the data used to create the boxplot, run the generalized linear mixed model, perform type III tests and perform pairwise comparisons of protection treatments.

Here,

Region and Location: the general region and the specific site, respectively

Plot_ID: individual nest plot

Treatment: the applied protection treatment

No_nest: the number of nests in given plot

Destroyed: the number of destroyed nests at the end of the burial period

Intact: the number of intact nests at the end of the burial period

%Destroyed and prop.destroyed: percentage and proportion of nests destroyed in a given plot, respectively

Start: date of nest plot creation

Finish: date of nest plot creation

Burial_weeks: total number of weeks nests were buried

**Sheet 2: Predator type data**

This was the data set used for the NMDS plots and PERMANOVA. Here, the column names are the same as above, only the individual predators are listed, as well as the corresponding proportion of nests in a given nest plot they were responsible for destroying.
